# Stent Failure Management in Contemporary Clinical Practice

**DOI:** 10.3390/diagnostics15131709

**Published:** 2025-07-04

**Authors:** Iosif Xenogiannis, Charalampos Varlamos, Despoina-Rafailia Benetou, Vassiliki-Maria Dragona, Stefanos Vlachos, Christos Pappas, Fotios Kolokathis, Grigoris V. Karamasis

**Affiliations:** Second Department of Cardiology, Attikon University Hospital, National and Kapodistrian University of Athens, Chaidari, 12461 Athens, Greecestefanosvlachos@yahoo.gr (S.V.); grigoris.karamasis@gmail.com (G.V.K.)

**Keywords:** drug-eluting stents, drug-coated balloons, in-stent restenosis, stent thrombosis, stent failure, intravascular imaging

## Abstract

**Background:** Although contemporary stent technology has significantly evolved, a substantial number of patients present with stent failure (SF), the clinical expression of which is either in-stent restenosis (ISR) or stent thrombosis (ST). **Methods:** In this observational, single-center study, we aimed to compare the clinical characteristics, clinical presentation, angiographic findings and subsequent management of patients who underwent percutaneous coronary intervention (PCI) for SF, either ISR or ST, with patients who had PCI for de novo lesions. **Results:** Over a period of two years (September 2022–October 2024), 1120 patients underwent PCI, of whom 9% had SF. Of the 101 SF cases, the majority (76 cases, 75%) had ISR, while the rest (25 cases, 25%) had ST. Regarding baseline characteristics, patients who underwent PCI for SF had a higher incidence of diabetes mellitus (53% vs. 29%; *p* < 0.001), dyslipidemia (88% vs. 50%; *p* < 0.001) as well as prior coronary artery bypass grafting surgery (7.9% vs. 3.7%; *p* = 0.043), while they were less likely to be current smokers (33% vs. 52%; *p* < 0.001). SF PCI patients presented more frequently with unstable angina (17% vs. 8.9%; *p* = 0.010). A new stent was implanted in less than half of SF cases (i.e., stent implantation, 44% vs. 91%; *p* < 0.001). On the other hand, in the clinical setting of SF, drug-coated balloons (44% vs. 5.3%; *p* < 0.001) and plain balloon angioplasty (8.9% vs. 0.7%; *p* < 0.001) was applied more frequently compared with de novo lesions. Furthermore, the usage of cutting/scoring balloons and lithotripsy was significantly higher in the SF group (8.9% vs. 0.4% and 12% vs. 3%, respectively; *p* < 0.001 for both). Intracoronary imaging guidance was more commonly used in the SF group (33% vs. 13%; *p* < 0.001). Stent malapposition (44%) and neoatherosclerosis (67%) were the most common mechanisms of ST and ISR, respectively, as identified by intravascular imaging modalities. Finally, the success rates were comparable (96% vs. 98%; *p* = 0.150) between the two groups. **Conclusions:** Approximately one of ten patients underwent PCI because of the failure of a previously implanted stent. Use of intracoronary imaging is significantly higher in the clinical context of SF. While DES implantation remains the standard of practice for de novo lesions, DCBs are a popular alternative, especially for ISR cases. Interventional cardiologists who are involved in the treatment of SF cases should be familiar with interpreting intravascular imaging to guide the use of the adjunctive device required to ensure that optimal procedural results in SF cases are obtained.

## 1. Introduction

The application of modern stent platforms due to their material (the majority is made by cobaltium chromium or platinum chromium), thinner struts and biodegradable or more biocompatible durable polymer coating in combination with the use of potent antiplatelet agents in contemporary clinical practice have significantly reduced stent failure (SF) rates [1,2,3,4,5]. Nevertheless, there is still a low but not negligible rate of SF after the first year of percutaneous coronary intervention (PCI) that is estimated to be roughly around 2% per year [6]. In-stent restenosis (ISR) remains the most common reason for SF, with an estimated 5-year ISR rate of 9% to 12% for non-complex lesions [7,8]. Multiple risk factors for ISR have been recognized and can be separated into the following three broad categories: patient (i.e., diabetes mellitus, renal insufficiency and acute coronary syndrome [ACS]), angiographic (i.e., long lesions [>20 mm], small vessel diameter [<3 mm], bifurcation lesions and severe calcification) and procedural (stent underexpansion, fracture and bare metal stent [BMS]) factors [9]. Approximately 4–10% of PCIs are performed in the context of ISR, illustrating the burden of the disease that interventional cardiologists are called to address [10,11]. The pathophysiology of ST and ISR differs significantly from that of de novo lesions. The following two main mechanisms have been implicated in the pathogenesis of ISR: (1) biologic mechanisms that are expressed with (a) the development of neointimal hyperplasia concerning mostly previously implanted BMS and (b) neoatherosclerosis associated mostly with previously implanted DES, and (2) mechanical mechanisms represented mainly by stent underexpansion and, to a lesser extent, stent fracture [9]. Likewise, a variety of mechanisms have been identified regarding stent thrombosis (ST), such as stent malapposition, stent underexpansion, edge dissection, raptured neoatherosclerotic lesion, uncovered struts and coronary evaginations [12,13,14].

The use of intravascular imagining is of paramount importance for the recognition of the underlying mechanism of SF, while specialized therapeutic approaches are frequently implemented for the management of SF. Emerging devices, such as drug-coated balloons (DCBs), have been used as an alternative to DES implantation for the treatment of ISR, which remains the standard of practice [15]. A plethora of adjunctive devices, namely, cutting and scoring balloons, super-high-pressure balloons, rotablation, excimer laser, brachytherapy and intravascular lithotripsy, have also been applied for the treatment of ISR [9,16]. In the present paper we examine the clinical characteristics, clinical presentation, angiographic findings and management of SF PCI cases in comparison with the PCI of de novo lesions, using contemporary data from a single university hospital, with the aim of identifying potential differences in the clinical presentation, intravascular imaging penetration, treatment techniques and devices used between the two groups. We also sought to recognize the underlying pathogenetic mechanisms of SF/ISR using intravascular imaging. 

## 2. Methods

We included 1120 consecutive patients hospitalized in the Second Department of Cardiology, Attikon University Hospital, Athens, Greece, who underwent PCI from September 2022 to October 2024. Patients with chronic total occlusions were excluded from the analysis. Patients were divided into the following two groups: those who underwent PCI for SF (either ISR or ST) and those who underwent PCI for de novo lesions. ISR was defined as the presence of (1) a stenosis causing a reduction of ≥50% of the luminal diameter within the previously stented segment, or within 5 mm proximal or distal from the edges of the previously implanted stent, accompanied by one of the following: (a) a positive history of recurrent angina pectoris, presumably related to the target vessel; (b) objective signs of ischemia at rest (ECG changes) or during a stress test presumably related to the target vessel; (c) abnormal results of any invasive functional diagnostic test; (2) a diameter stenosis ≥70% (with the angiographic characteristics described above) even in the absence of any ischemic signs or symptoms [16,17]. ST was defined as the angiographic presence of thrombus that originates in the stent or in the segment 5 mm proximal or distal to the stent, and the presence of at least one of the following criteria within a 48 h time window: (a) acute onset of ischemic symptoms at rest, (b) new ischemic electrocardiographic changes that suggest acute ischemia or (c) cardiac biomarker dynamic changes compatible with spontaneous myocardial infarction [17]. Unstable angina was defined as any of the following: (a) angina at rest lasting at least 20 min; (b) recent worsening (crescendo angina) that becomes more frequent, caused by less physical exertion than previously, lasts longer and is at least Canadian Classification Society class III; (c) new onset (de novo) within three months, with effort angina (class II or III of the Canadian Cardiovascular Society classification), and no troponin values > 52 pg/mL (Roche hs-troponin T was used in all cases). For the definition of myocardial infarction, we used the fourth universal definition of myocardial infarction [18]. Second-generation, including everolimus-, zotarolimus- and sirolimus-eluting, stents were used in both groups.

We compared clinical and technical characteristics, as well as procedural outcomes, between the two patient groups. Technical success was defined as the achievement of <30% residual diameter stenosis post-PCI within the treated vessel or stented segment, and restoration of TIMI grade 3 antegrade flow.

Categorical variables were expressed as percentages and were compared using Pearson’s chi-square test or the 2-tail Fisher’s exact test. Continuous variables were presented as mean ± standard deviation or median (interquartile range, IQR) and were compared using the *t*-test or Wilcoxon rank-sum test, as appropriate. All statistical analyses were performed with SPSS version 25.0 (Statistical Package for Social Sciences, IBM). A two-sided *p*-value of 0.05 was considered statistically significant.

## 3. Results

In total, 101 patients were included in the SF group, accounting for the 9% of total PCIs, and 1019 in the non-stent failure group (non-SF group). The major cause of SF was ISR (76 cases; 75%), while the rest was ST (25 cases; 25%). The majority of ISR cases (59/76, 78% vs. 17/76; 22%) occurred ≥ 1 year from the index stent implantation. With respect to the ST classification, the rates were as follows: (a) acute ST (<24 h), 1/25 (4%); (b) subacute ST (24 h to 1 month), 5/25 (20%); (c) late ST (1 month to 1 year), 4/25 (16%); (d) very late ST, 15/25 (60%).

Patients’ clinical characteristics are outlined in Table 1. Patients in the SF group were on average 3 years older while patients’ gender was similar between the two groups. In addition, patients in the SF group were less likely to be current smokers compared with patients in the non-SF group (33% vs. 52%; *p* < 0.001), whereas diabetes mellitus and dyslipidemia were more prevalent in the former group (53% vs. 29%, *p* < 0.001, and 88% vs. 50%, *p* < 0.001, respectively). Furthermore, patients in the SF were more like to have undergone previous coronary artery bypass graft surgery (7.9 % vs. 3.7%; *p* = 0.043).

**Figure 1:**
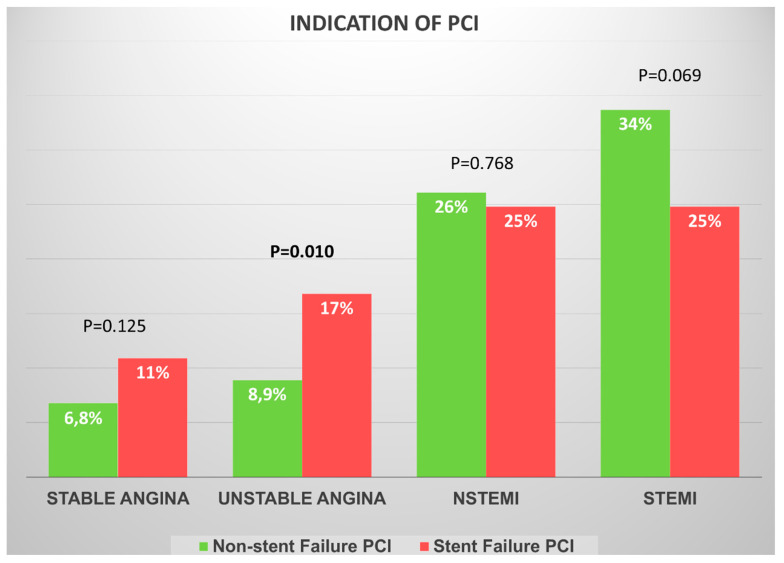
Clinical presentation according to the indication for percutaneous coronary intervention. NSTEMI, non-ST-segment elevation myocardial infarction; PCI, percutaneous coronary intervention; STEMI, ST-segment elevation myocardial infarction.

Clinical presentation is illustrated in Figure 1. The most common indication for both groups was ST-segment elevation myocardial infarction (STEMI) (25% vs. 34%; *p* = 0.069), while patients in the SF group presented more frequently with unstable angina in comparison to the non-SF group patients (17% vs. 8.9%; *p* = 0.010).

Angiographic and procedural characteristics are outlined in Table 2. The radial artery was more commonly used as the access site in the non-SF group (89% vs. 95%; *p* = 0.016). The circumflex artery was less frequently the target vessel for the SF group compared with the non-SF group (5.9% vs. 18%; *p* = 0.002), and it was more likely for the patients in the SF group to undergo multivessel PCI (14% vs. 5.8%; *p* = 0.001). Finally, specialized plaque modification devices, such as intravascular lithotripsy and cutting/scoring balloons, were utilized in a higher percentage in the SF group (12% vs. 3%, *p* < 0.001, and 8.9% vs. 0.4%, *p* < 0.001, for lithotripsy and cutting/scoring balloons, respectively).

**Figure 2:**
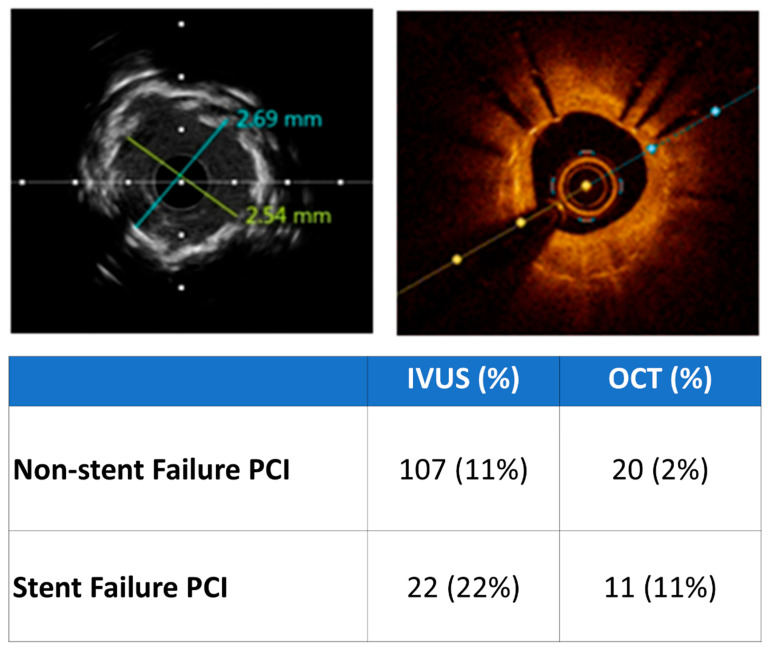
Intravascular coronary imaging penetration according the indication for percutaneous coronary intervention. IVUS, intravascular ultrasound; OCT, optical coherence tomography.

**Figure 3:**
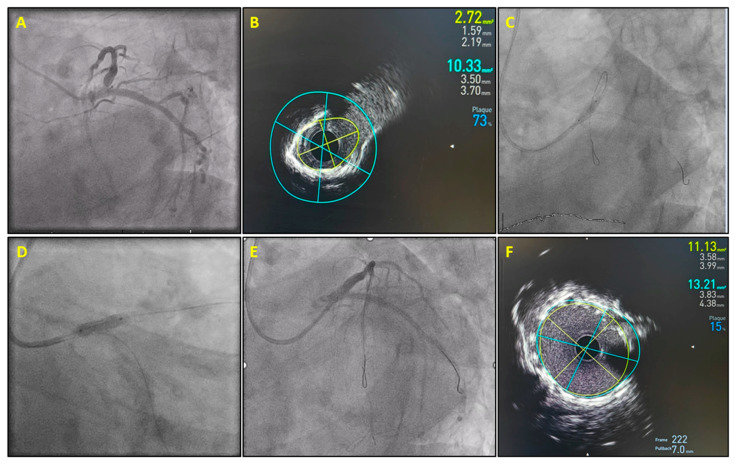
A 73-year-old woman presented complaining of exertional dyspnea. She had undergone left main percutaneous coronary intervention three years earlier. Coronary angiography showed an 80–90% focal stenosis in the ostium of the left anterior descending coronary artery inside the previously implanted stent (**A**). Intravascular ultrasound (IVUS) showed concentric calcification with underexpansion of the old stent (minimum stent area 2.7 mm^2^) (**B**). Inflations with a 4.0 mm non-compliant balloon failed to adequately expand the stent. Intravascular lithotripsy using a 4.0 mm balloon modified calcium and allowed subsequent symmetrical non-compliant balloon expansion (**C**). A 4.0 × 12 mm Megatron (Boston Scientific, Marlborough, MA, USA) drug-eluting stent was implanted (**D**). Final coronary angiography showed an excellent angiographic result (**E**), which was confirmed by IVUS imaging (minimum stent area, 9.1 mm^2^) (**F**).

Intracoronary imaging (ICI) guidance was applied almost three times more frequently in the SF group (33% vs. 13%; *p* < 0.001), as shown in Figure 2. Intravascular imaging rates were higher when PCI was performed in the left main (70%) or left anterior descending coronary artery (58%). Intravascular ultrasound (IVUS)-guided PCI was used in the majority of both de novo lesion (84% IVUS vs. 16% OCT) and SF cases (67% IVUS vs. 33% OCT), as shown in Figure 3. The most common mechanism was stent malapposition (44%) and the development of neoatherosclerosis (67%, including cases where both stent atherosclerosis and stent underexpansion were identified) for ST and ISR, respectively (Table 3 and Table 4).

**Figure 4:**
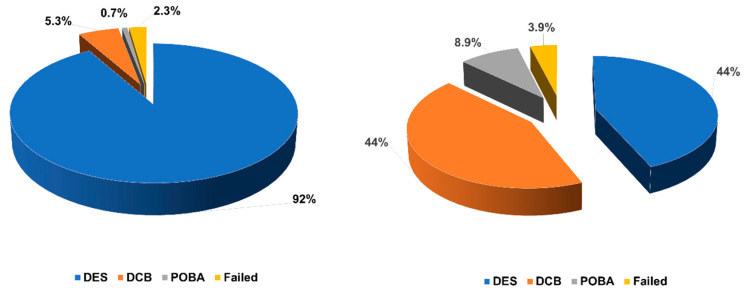
Treatment modality according to the indication for percutaneous coronary intervention. DES, drug-eluting stent; DCB, drug-coated balloon; POBA, plain old balloon angioplasty; PCI, percutaneous coronary intervention.

A new DES was implanted in less than half of patients (44%) with SF, while stent implantation was by far the commonest PCI mode in the non-SF group (44% vs. 91%; *p* < 0.001), as shown in Figure 4. DCB angioplasty was used in a large portion of patients with SF, whereas it was applied only in a small minority of patients with de novo lesions (44% vs. 5.3%; *p* < 0.001). Finally, successful PCI rates were similar between the two groups (96% vs. 98%; *p* = 0.150). 

Antithrombotic regiments at discharge for the two SF subgroups were as follows: regarding ISR patients, 46% of them discharged on aspirin and ticagrelor; 30% on aspirin, clopidogrel plus new oral anticoagulant (NOAC); 22% on aspirin and clopidogrel; and 2% on aspirin and prasugrel. The vast majority of patients with ST were discharged on aspirin and ticagrelor (84%), while 11% and 5% were discharged on aspirin, clopidogrel plus NOAC and aspirin, and clopidogrel plus acenocumarol, respectively.

## 4. Discussion

The main findings of our study are the following: (1) Approximately 9% of the total number of PCIs were performed because of a prior SF; (2) ICI was performed in one of three patients presenting with SF, and significantly more frequently compared with de novo lesions; (3) while stent implantation was performed in the vast majority of non-SF cases, a new stent was used in less than 50% of patients with SF, with DCB being a popular alternative in the latter group; (4) there was no statistically significant difference in the rate of successful PCI between the SF and non-SF cases. 

Our findings regarding the incidence of ISR PCI are in concordance with previous studies reporting that ISR PCI accounts for 4–10% of total PCIs [7,10,11,19]. Interestingly, according to the reports from European registries, ISR PCI rates are constantly lower, reaching roughly 5% [7,11], in comparison with data derived from American observational studies, where the corresponding rate is 10% [10,19].

In a study by Moussa et al., approximately 27% of patients presented with MI (8.5% STEMI and 18.7% with non-STEMI) and another 50% with unstable angina [10]. Likewise, Tamez et al. reported that 67% of patients with ISR underwent PCI for an ACS (MI or unstable angina), illustrating that ISR is far from being considered as a benign clinical entity [19]. Furthermore, Waldo et al., using data derived from the Veterans Affairs (VA) Clinical Assessment and Tracking (CART) Program, found that 20% of patients diagnosed with ISR had MI (3% STEMI and 17% non-STEMI), and 33% unstable angina at initial presentation [20]. The low percentage of STEMI in this cohort could be explained by the fact that it is likely that veterans with STEMI were transferred to centers outside VA hospital system for primary PCI [10]. The rates of ACS as the clinical indication for ISR PCI were similar in a recent publication by the Korean Stent Failure Research (SFR) group DCB registry, accounting for 62% of total ISR PCIs (5% STEMI, 20% non-STEMI and 38% unstable angina), where, once again, the majority of patients with ISR presented with unstable rather than stable coronary artery disease [21]. The higher percentage of MI (24% STEMI and 17% non-STEMI) in our study could be justified by the fact that a considerable proportion of our patients presented with ST rather than ISR. Indeed, ST is clinically expressed in up to 80–90% as STEMI, and is associated with mortality rates as high as 50% [14,22,23].

Both the American as well as the European Guidelines encourage the use of ICI in cases of SF (class IIa, level of evidence C) for the clarification of the underlying mechanism [24,25]. However, ICI remains underused in clinical practice. In the landmark randomized controlled trials (RCTs) on the management of ISR, ICI was not mandatory and the rate of utilization is not reported [26,27,28]. The same is evident for large retrospective studies [10,21]. In the study by Waldo et al., IVUS utilization was only 15%, whereas there is no reference concerning OCT usage [20]. In our study, the ICI application rate was more than two times higher. A possible explanation is that the population included in the previously mentioned study was recruited from 2006 to 2014. In the meanwhile, RCTs provided evidence for the superiority of ICI-guided PCI vs. angiography-guided PCI, enforcing the position of ICI in contemporary PCI [29,30]. Nevertheless, until now, there have been no dedicated RCTs evaluating the usefulness of ICI in the clinical context of ISR PCI. The Randomized Controlled Trial of Intravascular Imaging Guidance versus Angiography-Guidance on Clinical Outcomes after Complex Percutaneous Coronary Intervention (RENOVATE-COMPLEX-PCI) and Optical Coherence Tomography (OCT) Guided Coronary Stent Implantation Compared with Angiography: A Multicenter Randomized Trial in PCI (ILUMIEN IV: OPTIMAL PCI) compared ICI (the former either IVUS or OCT, and the latter only OCT) with angiography-PCI guidance [31,32]. In the subgroup analysis of both trials, the use of ICI did not reduce the incidence of the primary end point of target vessel failure in the clinical setting of ISR. Nevertheless, those trials were not powered for patients with ISR. Karamasis et al. reported that ICI utilization in ST cases had an impact on subsequent management (stent vs. non-stent) based on the underlying pathophysiological mechanism. Furthermore, ICI-guided management increased survival free of death and target lesion revascularization [33]. Multiple factors might play a role regarding the low penetrance of intravascular imaging modalities in cases of SF: availability, increased cost and time of operation, lack of training as well as the absence of randomized data supporting improved clinical outcomes with the use of IVUS/OCT in the context of SF.

Shlofmitz et al. proposed a new classification for ISR based on the cause of SF, proposing the following five main types: mechanical (type I), biologic (type II), mixed pattern (type III), chronic total occlusion (type IV) and > two stent layers (type V) [34]. The investigators provided therapeutic guidance according to the type of ISR. In our study, we were able to identify the mechanism of ISR and ST in all cases where intravascular imaging was applied. Given the excellent performance of intravascular imaging in recognizing the underlying pathogenetic mechanism of SF, and in accordance with the recommendations of contemporary guidelines, we believe in the default usage of intravascular imaging in cases of SF, since the recognition of the SF cause guides subsequent interventional treatment, thus improving potentially clinical outcomes. Hence, the training of interventional cardiologists to recognize different SF patterns by IVUS/OCT is paramount.

The implantation of a DES has been the mainstay strategy for the treatment of SF, used in 73–83% of the ISR cases in the USA [10,19]. DCBs are an appealing alternative, since they provide an antiproliferative drug to the vessel segment where ISR occurs, without the need for the placement of a new scaffold. Interestingly, DCBs were only recently approved for use in coronary interventions in the USA, explaining the lower rates of DES implantation in our registry, since DCBs were already available for use in our country. The superiority of DCBs over POBA, in terms of MACE, has been shown in multiple RCTs [35,36]. According to the results of the Difference in Antirestenotic Effectiveness of Drug-Eluting Stent and Drug-Coated Balloon Angioplasty for the Occurrence of Coronary In-Stent Restenosis (DEDALUS) study, which was a pooled analysis of individual patient data from ten RCTs comparing the effectiveness of DCBs vs. DES for the treatment of ISR, DES were more effective in reducing target vessel revascularization at three years compared with DCBs in the presence of a restenotic DES, while there was no significant difference between the two treatments in bare-metal stent (BMS)-ISR [37]. The DEDALUS study included trials where paclitaxel-eluting (in three trials, not available anymore), everolimus-eluting (in six trials) and sirolimus-eluting (in one trial) stents had been used for the treatment of ISR. In another metanalysis, which included only trials where everolimus-eluting stents had been used, DES were superior to DCBs with respect to targe vessel revascularization at three years for any ISR (DES or BMS) [38]. Given the previous, the recent European Guidelines encourage the use of DES over DCBs for treatment of in-DES restenosis (class I, level of evidence A), while they do not give any specific recommendation for BMS-ISR where DCBs seem to be equally effective [39]. It is worth noting that, although sirolimus-coated DCBs are available in the market, only paclitaxel-coated DCBs have been investigated in RCTs in the setting of ISR. Meticulous lesion preparation is of great significance before the application of DCBs, since the utilization of scoring balloons, before PCI with DCB, reduces the rate of restenosis, likely because of better drug absorption [40]. In summary, although DCBs have the advantage of leaving no metallic material behind after vessel dilatation, thus avoiding the risk of restenosis inside the new scaffold, they have worse long-term clinical outcomes compared with everolimus DES in the clinical setting of ISR. In addition, they should not be used for the mechanical treatment of ISR, for example, in cases of underexpansion due to severe calcification or stent recoil, as they are not meant to provide adequate stent expansion, but rather to deliver drug after optimal dilatation [41]. Thus, they can only be applied after appropriate vessel preparation with conventional or/and cutting scoring balloons, when a good angiographic result has been achieved without major dissections that compromise the flow to < TIMI flow III and in the absence of > 30% residual stenosis [41]. Lithotripsy has been used to adequately expand previously implanted, underexpanded stents that had been placed over not appropriately prepared calcified lesions. In addition, lithotripsy has been associated with less residual stenosis compared to cutting/scoring balloons in the clinical context of ISR [42]. Taking under consideration the previous, the higher percentage of cutting balloons and lithotripsy use in our study seems justified. With respect to the findings of our study, an equal percentage of patients with SF were treated with new DES and DCBs, while intravascular lithotripsy was applied in more than one in ten patients, and scoring/cutting balloons in 9% of patients with SF, significantly more frequently compared with de novo lesion cases. Given the previous, we strongly believe that the previously mentioned specialized balloons/devices should be readily available, and the operators have to be familiar with their use when they are involved in interventions for SF.

Data regarding the management of ST remain scarce. ICI can delineate the underlying pathophysiological mechanism (most commonly stent malapposition/uncovered struts, stent underexpansion, edge dissection and ruptured neoatherosclerosis) and guide management [14]. It is estimated that a new stent is implanted in approximately 45–64% of cases [43,44,45,46]. However, the effect of repeat stent implantation in ST regarding mortality remains contradictory, and the role of DCBs is largely unknown [23,43,44,45]. Although the majority of these studies showed that repeat stent implantation was associated with higher mortality, they are outdated, as they used old generation stents, while the most contemporary study that used new generation DES in half of patients reported higher survival for those who underwent repeat stenting [23]. Furthermore, it is likely that there is a bias towards deferring stent placement in more complex lesions that led to ST (i.e., calcified long or tortuous lesions). According to a recent SCAI expert consensus statement on the management of ISR and ST, additional stent implantation should be limited to cover residual edge dissections, while the cornerstones of treatment are the dilatations of non-compliant/high-pressure balloons and intravascular lithotripsy when needed, aspiration thrombectomy and potent antithrombotic agents [9].

Our study has several limitations. It is an observational study, and thus subject to bias. The population of the study is not well-balanced between the two groups, and rather small regarding the SF group. Additionally, we do not have any data regarding the type of the failed stents and the clinical indication for the index PCI. Furthermore, we are unaware about the rates of usage of intravascular imaging during the index procedure and, thus, the impact that the upfront intravascular imaging utilization would have made on reducing SF rates. Finally, we did not collect data concerning medication compliance of the SF patients.

## 5. Conclusions

Although the technology of new DES has evolved, PCI in one of ten patients is performed because of a previously implanted failed stent. ICI is used more often in this clinical scenario in order to recognize the mechanism of ISR/ST, guide therapy and prevent repeat failure. While DES implantation remains the standard of practice for de novo lesions, DCBs are a popular alternative, especially for ISR cases. Interventional cardiologists who are involved in the treatment of SF cases should be familiar with the interpretation of the patterns of failure, as they are imaged by intravascular imaging modalities as well as with the use of adjunctive devices, such as scoring/cutting balloons and intravascular lithotripsy, for achieving optimal angiographic results.

## Figures and Tables

**Table 1:** Patients’ clinical characteristics. The *p*-values concern the no-stent failure vs. stent failure groups. No comparison was made for in-stent restenosis vs. stent thrombosis.

Variables	Non-Stent Failure (*n* = 1019)	Stent Failure (*n* = 101)	In-Stent Restenosis (*n* = 76)	Stent Thrombosis (*n* = 25)	*p*-Value
Mean age (years)	65	68	68	65	**0.031**
Female gender (%)	224 (22%)	16 (16%)	12 (16%)	4 (16%)	0.151
Current smoker (%)	532 (52%)	33 (33%)	21 (28%)	12 (48%)	**<0.001**
Diabetes mellitus (%)	296 (29%)	53 (53%)	43 (57%)	10 (40%)	**0.001**
Arterial hypertension (%)	577(57%)	64 (63%)	51 (67%)	13 (52%)	0.191
Dyslipidemia (%)	511 (50%)	89 (88%)	67 (88%)	22 (88%)	**<0.001**
Family history of CAD (%)	143 (14%)	6 (5.9%)	3 (3.9%)	3 (12%)	**0.022**
Atrial fibrillation (%)	72 (7.1%)	11 (11%)	9 (12%)	2 (8%)	0.162
Prior CABG (%)	38 (3.7%)	8 (7.9%)	8 (11%)	0 (0.0%)	**0.043**

CAD, coronary artery disease; CABG, coronary artery bypass graft surgery.

**Table 2:** Angiographic and procedural characteristics. The *p*-values concern no-stent failure vs. stent failure groups. No comparison was made for in-stent restenosis vs. stent thrombosis.

Characteristics	Non-Stent Failure (*n* = 1019)	Stent Failure (*n* = 101)	In-Stent Restenosis (*n* = 76)	Stent Thrombosis (*n* = 25)	*p*-Value
Radial access (%)	967 (95%)	90 (89%)	67 (88%)	23 (92%)	**0.016**
Vessel of PCI					
LM PCI (%)	79 (7.8%)	10 (9.9%)	6 (7.9%)	4 (16%)	0.446
LAD PCI (%)	419 (41%)	43 (43%)	31 (41%)	12 (48%)	0.777
LCX PCI (%)	181 (18%)	6 (5.9%)	5 (6.6%)	1 (4%)	**0.002**
RCA PCI (%)	267 (26%)	27 (27%)	21 (28%)	6 (24%)	0.908
Multivessel PCI (%)	59 (5.8%)	14 (14%)	12 (16%)	2 (8%)	**0.001**
Graft PCI (%)	14 (1.4%)	1 (1.0%)	1 (1.3%)	0 (0.0%)	1.000
Mean stent length per patients (mm)	31	30.8	32.2	25.3	0.873
Largest stent mean diameter (mm)	3.21	3.24	3.23	3.31	0.639
Utilization of balloons/adjunctive devices					
NC balloon (%)	446 (44%)	72 (71%)	53 (70%)	19 (76%)	**<0.001**
Cutting/scoring balloon (%)	4 (0.4%)	9 (8.9%)	8 (11%)	1 (4%)	**<0.001**
Lithotripsy (%)	31 (3.0%)	12 (12%)	12 (16%)	0 (0%)	**<0.001**
Rotablation (%)	12 (1.2%)	1 (1%)	1 (1.3%)	0 (0%)	1.000
Technical success (%)	996 (98%)	97 (96%)	72 (95%)	25 (100%)	0.296

LAD, left anterior descending; LM, left main; LCX, left circumflex; NC, non-compliant; PCI, percutaneous coronary intervention; RCA, right coronary artery.

**Table 3:** Mechanisms of stent thrombosis as they were identified by optical coherence tomography or intravascular ultrasound according to the operator.

	Malapposition	Raptured Neoathersoclerotic Plaque	Underexpansion	Stent Edge Dissection
OCT (6)	3 (50%)	1 (17%)	2 (33%)	1 (17%)
IVUS (3)	1 (33%)	2 (66%)	0	0
Total (9)	4 (44%)	3 (33%)	2 (22%)	1 (11%)

OCT, optical coherence tomography; IVUS, intravascular ultrasound. Note: in one case were identified two potential mechanisms of stent thrombosis.

**Table 4:** Mechanisms of in-stent restenosis as they were identified by optical coherence tomography or intravascular ultrasound according to the operator.

	Neoatherosclerosis	Underexpansion	Underexpansion and Neoatherosclerosis
OCT (5)	3 (60%)	1 (20%)	1 (20%)
IVUS (19)	9 (47%)	7 (37%)	3 (16%)
Total (24)	12 (50%)	8 (33%)	4 (17%)

OCT, optical coherence tomography; IVUS, intravascular ultrasound.

## Data Availability

The datasets presented in this article are not readily available. However, requests to access the datasets can be directed to the corresponding author and will be examined.
